# Development of a framework to improve the process of recruitment to randomised controlled trials (RCTs): the SEAR (Screened, Eligible, Approached, Randomised) framework

**DOI:** 10.1186/s13063-017-2413-6

**Published:** 2018-01-19

**Authors:** Caroline Wilson, Leila Rooshenas, Sangeetha Paramasivan, Daisy Elliott, Marcus Jepson, Sean Strong, Alison Birtle, David J. Beard, Alison Halliday, Freddie C. Hamdy, Rebecca Lewis, Chris Metcalfe, Chris A. Rogers, Robert C. Stein, Jane M. Blazeby, Jenny L. Donovan

**Affiliations:** 1 School of Social and Community Medicine, University of Bristol, 39 Whatley Road, Bristol, BS8 2PS UK; 2 Rosemere Cancer Centre, Royal Preston Hospital, Sharoe Green Land North, Fulwood, Preston, Lancashire PR2 9HT UK; 3 Nuffield Department of Orthopaedics, Rheumatology and Musculoskeletal Sciences, University of Oxford, Oxford, OX3 7LD UK; 4 Nuffield Department of Surgical Sciences, University of Oxford, Oxford, OX3 9DU UK; 5 Institute of Cancer Research Clinical Trials and Statistics Unit (ICR-CTSU), Institute of Cancer Research, 15 Cotswold Road, Sutton, SM2 5NG UK; 6 Bristol Randomised Trials Collaboration University of Bristol, School of Social and Community Medicine, 39 Whatley Road, Bristol, BS8 2PS UK; 7 Clinical Trials and Evaluation Unit, School of Clinical Sciences, University of Bristol, Level 7 Queens Building, Bristol Royal Infirmary, Bristol, BS2 8HW UK; 8 NIHR University College London Hospitals Biomedical Research Centre, 149 Tottenham Court Road, London, W1T 7DN UK; 9 Collaboration for Leadership in Applied Health Research and Care West, University Hospitals Bristol, 9th Floor, Whitefriars Lewins, Bristol, BS1 2NT UK

**Keywords:** Recruitment, Randomised controlled trials, Screening, Screening logs, Eligibility assessment, Non-participation, CONSORT statement

## Abstract

**Background:**

Research has shown that recruitment to trials is a process that stretches from identifying potentially eligible patients, through eligibility assessment, to obtaining informed consent. The length and complexity of this pathway means that many patients do not have the opportunity to consider participation. This article presents the development of a simple framework to document, understand and improve the process of trial recruitment.

**Methods:**

Eight RCTs integrated a QuinteT Recruitment Intervention (QRI) into the main trial, feasibility or pilot study. Part of the QRI required mapping the patient recruitment pathway using trial-specific screening and recruitment logs. A content analysis compared the logs to identify aspects of the recruitment pathway and process that were useful in monitoring and improving recruitment. Findings were synthesised to develop an optimised simple framework that can be used in a wide range of RCTs.

**Results:**

The eight trials recorded basic information about patients screened for trial participation and randomisation outcome. Three trials systematically recorded reasons why an individual was not enrolled in the trial, and further details why they were not eligible or approached, or declined randomisation. A framework to facilitate clearer recording of the recruitment process and reasons for non-participation was developed: SEAR – Screening, to identify potentially eligible trial participants; Eligibility, assessed against the trial protocol inclusion/exclusion criteria; Approach, the provision of oral and written information and invitation to participate in the trial, and Randomised or not, with the outcome of randomisation or treatment received.

**Conclusions:**

The SEAR framework encourages the collection of information to identify recruitment obstacles and facilitate improvements to the recruitment process. SEAR can be adapted to monitor recruitment to most RCTs, but is likely to add most value in trials where recruitment problems are anticipated or evident. Further work to test it more widely is recommended.

## Background

Randomised controlled trials (RCTs) are regarded as the most reliable and effective method to evaluate healthcare interventions. However, many RCTs struggle to recruit to target and to time, leading to underpowered studies, costly extensions or the early closure of studies [1–3]. Qualitative research has shown that the process of recruitment can be complex, protracted and fragile [4, 5]. The Qualitative research Integrated within Trials (QuinteT) Recruitment Intervention (QRI) uses standard and innovative qualitative research methods and simple quantification techniques to understand the recruitment process, and identify and address challenges as they emerge [6]. An integral part of the QRI involves mapping the pathway to recruitment for potential participants, to better understand barriers to recruitment across the trial and between clinical centres in multicentre trials.

Maintaining an accurate record of patients considered for RCT participation is a recommendation in Consolidated Standards of Reporting Trials (CONSORT) reporting guidelines. Trials should present the numbers assessed for eligibility, and excluded because they did not meet the inclusion criteria, they declined to take part, or ‘other reason’. [7]. The CONSORT flowchart does not however, explicitly represent all the steps in the pathway that potential RCT participants can follow. Furthermore, reviews of published RCT results have shown that trials consistently fail to record participant flow accurately, particularly before informed consent and randomisation [3, 8].

The collection of screening data is also a consideration under Good Clinical Practice (GCP), in particular to monitor compliance with the trial protocol inclusion and exclusion criteria [9]. However, the literature on screening logs is sparse and there is little consensus about what to collect or how to collect it efficiently. As the QRI includes analysis of data collected during screening and eligibility assessment, we investigated the range of data collected in eight RCTs with the aim of developing a simple framework that could be applied to most trials, to provide basic information useful to understand recruitment challenges and improve the recruitment process.

## Methods

All trials which had worked with the QuinteT researchers to optimise recruitment and informed consent were considered for inclusion (*n* = 14). Six trials considered in previous development work to map the recruitment pathway were excluded [4, 5, 10]. Eight trials anticipating or experiencing recruitment difficulties were included using a convenience sampling approach. The eight RCTs were managed by seven different clinical trials units (CTUs); three were feasibility studies, two pilot studies and three main trials. Ethical approval for the QRI research, including the use of screening log data, was obtained within the governance arrangement for each RCT.

We use the term ‘screening’ or ‘recruitment’ log to refer to an “essential document that records all individuals who entered pre-screening or screening, and details the reasons why an individual is not enrolled ” [11]. Following this definition, we focused on two functions of a screening log:

1. to record key characteristics of all individuals considered for trial participation
2. to capture the reasons *why* an individual was not enrolled in a trial

We conducted a directed content analysis [12] of the screening logs used in the eight trials, to identify which aspects of the recruitment pathway and process were assessed and which were useful to monitor recruitment. We reviewed all trial documentation [protocol, manual, case report forms (CRFs), screening logs] and extracted all references to recruitment, screening, screening log, eligibility checks, information provision, and randomisation. Data were analysed thematically and synthesised using the constant comparative method, adapted from grounded theory [13, 14].

As part of the thematic analysis, codes were developed to summarise the information included in each screening log. The codes were used to identify the recruitment stages. The thematic analysis was conducted independently by the first author (CW) and the trial-specific QuinteT researcher (DE, MJ, SP, LR, and SS). The codes were presented to the wider group (DE, MJ, SP, LR, SS, CW, and JD) to develop a consensus about the key stages in the recruitment process that could be captured by a log. The stages (screening, eligibility assessment, approaching, and recruited/randomised) were then used to conduct a directed content analysis to identify how each trial recorded this information. The findings from the content analysis were further synthesised by CW to produce the SEAR framework. The recommendations presented in the article were developed by CW and JD, and are a synthesis of the findings from this study, combined with experience from other QRIs in RCTs addressing recruitment problems.

## Results

### Characteristics of RCTs and recruitment pathway

The eight RCTs were funded by major UK public funding bodies. Recruitment to all RCTs was NHS hospital-based and compared treatments for cancer (five trials) and other chronic health conditions (three trials). Six out of the eight trials included at least one surgical arm and four RCTs had a ‘no’ or ‘less’ treatment arm (Table 1). Recruitment to all trials was doctor-led with support from research nurses and other clinical staff. The recruitment target for the majority of trials was less than 350, with the exception of T5 and T6.

**Table 1:** RCT characteristics

	Clinical context	RCT type	Clinical centres (n)	Target recruitment figure (n)	Specialties involved	Intervention arms	Trial-specific recruitment pathway challenge
T1	Cancer treatment	Main	71	345	Surgery, oncology	Chemotherapy; surveillance (2)	Pre-trial treatment. Recruitment across surgery/ oncology. Alternative care pathway for trial. Multiple tests/ delays determining eligibility.
T2	Orthopaedics	Main	14	300	Surgery	Surgery; placebo surgery; active monitoring (3)	Patients referred from GPs for treatment X.
T3	Cancer	Feasibility	35	313	Surgery, oncology	Chemotherapy; test-directed chemotherapy (2)	Pre-trial treatment. Recruitment across surgery/ oncology.
T4	Cancer	Pilot	2	36	Surgery	Open surgery; minimally invasive surgery (2)	Pre-trial treatment. Recruitment across surgery/ oncology. Multiple tests/ delays determining eligibility.
T5	Vascular surgery/ interventional radiology	Main	18 (UK)	500–600 (UK) 3600 internationally	Surgery	Surgery; stenting (2)	Alternative care pathway for trial.
T6	Bariatric surgery	Internal pilot phase of a main RCT	2	89	Surgery	Banding; bypass surgery (2)	Multiple tests/delays determining eligibility.
T7	Cancer	Feasibility	2	Not set	Surgery, oncology	Surgery; radiotherapy (2)	Recruitment across surgery/ oncology. Alternative care pathway for trial.
T8	Cancer	Feasibility	5	50	Surgery	Radical surgery; partial surgery (2)	Multiple tests/ delays determining eligibility.

*GP* general practitioner; *RCT* randomised controlled trial

In all trials, the recruitment process was protracted due to tests to determine eligibility (all RCTs), the necessity of pre-trial treatment (T1, T3, T4), collaboration across medical specialisms (T1, T3, T5, T7), and/or new care pathways to recruit and treat RCT participants (T1, T5, T7). As part of the QuinteT Recruitment Intervention (QRI), all RCTs were encouraged to create and maintain comprehensive records of all individuals screened for trial participation to generate basic data about levels of eligibility and recruitment, and identify points at which potential participants opted in or out of the RCT, or were lost from the process.

All screening logs were designed by the RCT CTU, or trial manager (TM) and clinical lead (T4, T7 only). In general, the chief investigators (CI), trial managers and CTUs were committed to keeping screening records up to date, despite the time and effort required to do so. Research nurses in several trials reported informally that maintaining screening records was a low priority compared to other trial tasks. Trials with a particularly long or complex recruitment pathway, trials for rare conditions, pilot and feasibility studies showed greater commitment to keep screening records up to date, even though similar instructions were included in all trial protocols.

### Screening logs to map the recruitment pathway

All trial protocols included instructions to keep a record of all participants screened for trial participation. Screening records were mentioned as part of the QRI section of the protocol (T2, T5, T8) or in the main protocol or trial manual (T1, T3, T4, T6, T7). The most comprehensive description included a section on recruitment which outlined how patients should be identified and logged, and an explanation of the purpose of keeping up-to-date screening logs: to monitor and improve recruitment, assess compliance with the trial protocol, identify variations in recruitment between centres, and identify points where patients were ‘lost’ from the RCT (T1).

The majority of trials (except T6 and T7) requested information about potential trial participants on a ‘centre’ log – a landscape document, using one row to record between 10 and 15 pieces of information per screened participant, usually returned to the CTU every month, excluding any patient identifiable data (e.g. initials or hospital number), in accordance with GCP guidelines. Three trials (T4, T6, T7) used an ‘individual’ screening log to map the patient pathway, similar in style to a case report form (CRF). Anonymised data (excluding any patient identifiers) were input from the CRF form into a database to share information with the CTU. Individual screening logs recorded more information, between 22 and 68 pieces of data, per screened participant. An individual form was useful at multidisciplinary team (MDT) meetings for trials with complex inclusion/exclusion criteria, and when more extensive eligibility checks needed to be completed before randomisation. T4 used three forms, two centre screening logs (filled out pre and post pre-trial treatment) and an individual form to record further details about eligibility after pre-trial treatment. T1 also performed more detailed centralised eligibility checks on a CRF, filled out after participants had consented to be randomised, in addition to basic eligibility information recorded on the centre log.

### Mapping the recruitment pathway

Initially, the thematic analysis of the eight trials’ screening logs identified six codes to classify all data collected: screening instructions/number; patient identifiers/ demographic information; outcome of eligibility checks; approaching patients; final decision about trial participation; and substudy-related information. Of the six codes, four related to major stages of the recruitment pathway and process:

- Screening: the identification of potentially eligible participants and entry onto a screening or recruitment log
- Eligibility assessment: checks against essential inclusion/exclusion criteria in the trial protocol to establish suitability for the trial
- Approach: the provision of information about the trial and invitation to take part
- Randomised: randomisation outcome, or treatment chosen by those declining randomisation

Data requested during these four stages of the recruitment process are summarised in Table 2 and analysed in further detail below.

**Table 2:** Comparison of data recorded in the eight RCTs. Summary of data recorded using the SEAR framework

	Screened NB any patient identifiable data recorded on screening log is not shared with CTU	Eligible	Approached	Randomised?
T1	Patient initials, NHS number, age, gender, screening ID, date of qualifying intervention. *Screening criteria:* Yes. Complete for all patients referred for Treatment X	Ineligible recorded as final screening outcome Reason for ineligibility (open response) Histology, pathology	Date two written information sheets provided Non-approach recorded as final screening outcome Reason for non-approach (open response)	Randomisation ID and allocation If not randomised, reason for non-participation (open response)
T2	Patient initials, gender, date of potential recruitment. *Screening criteria:* No	Reason for exclusion or ineligibility (using coded inclusion and exclusion criteria printed on back of the log)	Not recorded	Reason for eligible patients declining the trial (6 codes) Final treatment recorded for all screened participants Treatment preferences for eligible decliners
T3	Patient initials, age, screening ID, date screening initiated. *Screening criteria:* Yes. Complete for all patients with whom the trial is discussed.	Clinical data – nodal status (+/), tumour size Was the patient eligible? (Y/N) Reason for ineligibility (open response)	Not recorded	Consent (Y/N) for main and two substudies and participant ID Reason to decline main study and two substudies (open response) Final treatment received for all screened participants
T4	Patient initials, screening ID, age range, gender, MDT date. *Screening criteria:* Yes. Complete for all patients referred for Treatment X	MDT decision – potentially eligible (Y/N) and reason (open response); eligibility checked against each inclusion/exclusion criteria; Final MDT decision eligible (Y/N). If not, reason recorded (coded and/or open response).	‘Reason not eligible’ includes 3 coded responses why patient not approached	Randomisation ID Reason eligible patients decline trial (open response) Treatment received by non-randomised patients
T5	Patient initial, gender, age, hospital ID, referral from (5 codes). *Screening criteria:* No.	Eligible (Y/N)	Written information given (Y/N) Date trial discussed	Randomisation ID Patient consent (Y/N). If no, record reason Final treatment decision (non-randomised patients)
T6	Patient name, study ID, hospital number. *Screening criteria*: Complete for all patients referred for intervention X. Kept on site form – not entered into database if patient does not join the trial.	Eligibility (Y/N) against all trial inclusion/ exclusion criteria	Written information given (Y/N), date provided, reason not given (open response) Date of trial discussed Approached to participate in trial (Y/N) and reason not approached (open response) Coded when added to the study database	Reason eligible patient declined consent Final treatment decision (non-randomised patients) Coded when added to the study database
T7	Patient initials, date of birth, study number, gender, date of MDT decision, pathology. *Screening criteria:* Yes. 3 ‘initial’ trial eligibility criteria	Eligibility against all trial inclusion/exclusion criteria (Y/N). Reason for ineligibility (open response)	Patient invite to join study (Y/N). Reason (2 codes/open response)	Reason eligible patient declined trial (open response) Final treatment received (non-randomised patients)
T8	Patient initials, NHS no., date first screened. *Screening criteria*: Please enter all patients recommended by MDT as potentially eligible for Trial X	Inclusion/exclusion criteria met (Y/N) Reason for ineligibility	Provision of written information (Y/N) Approach consent for trial (Y/N)	Consent given (Y/N) Randomised (Y/N) Reason eligible patients declined (open response)

*CTU* Clinical Trials Unit, *MDT* multidisciplinary team, *NHS* National Health Service, *RCT* randomised controlled trial, *SEAR* Screened, Eligible, Approached, Randomised

#### Screening

This part of the process varied considerably between trials. When potential participants were first identified and entered onto the screening log (Screening), the majority of trials recorded a date early in the recruitment pathway, patient data (initials, gender, age, hospital ID, kept locally), and a screening number. Three trials (T2, T5, T8) did not assign screening numbers as they had a relatively straightforward pathway from initial identification to the results of eligibility checks.

Half the trials (T1, T4, T6, T7) had straightforward screening criteria, for example that all participants referred for treatment X were potentially eligible. In general, these trials were able to screen and conduct basic eligibility checks on all potential participants referred for treatment. T7 included three ‘initial trial eligibility’ criteria in the protocol – ‘histology evidence of condition X; no evidence of condition Y; fit for treatment’ to guide MDT decision-making. Several trials (T2, T3, T8) described a ‘potentially eligible participant’, (e.g. a person diagnosed with condition X, fit for treatment Y), which preceded the list of inclusion and exclusion criteria in the trial protocol. Three trial protocols left more scope for clinical judgement to identify individuals to screen (T2, T3, T5). For example,

“Patients likely to require surgery X for condition Y will be identified in outpatient clinics.” (T2)

“The screening log is to be filled out for all patients considered for trial participation but subsequently excluded.” (T3)

However, on the actual screening log, the instructions were to only enter individuals with whom the trial was discussed (T3):

“Please fill out details below for ALL patients with whom T3 is **discussed**. Include patients who turn out to be ineligible, and those who decline to consent.” *(Original emphasis)*

One trial had no screening instructions or criteria, only a description of eligibility, based on clinical judgement alone (T5):

“Eligibility: Patient has X that is thought to need some procedural intervention. Test shows intervention A and intervention B are both anatomically practicable, but both doctor and patient are substantially uncertain whether intervention A or intervention B is preferable.”

#### Eligibility

All trials (except T5) included a comprehensive list of inclusion and exclusion criteria in the trial protocol to determine participant eligibility or exclusion. The majority of inclusion and exclusion criteria were based on clinical findings which would affect the suitability of an individual to benefit from the treatments under investigation. In T4, the term ‘ineligible’ was used to code a range of clinical and non-clinical factors for non-participation (e.g. different histology; logistical reasons (unable to provide treatment within a specified time frame); unable to be approached (too ill), patient preferences (patient refused).

All screening logs recorded the final outcome of eligibility checks, using four different methods: Yes/No (T5), Yes/No plus open text box to explain reason for ineligibility (T3, T4, T8), a checklist against each inclusion/exclusion criterion and final outcome (T4, T6, T7), or ineligibility, as a final outcome of the screening process (T1, discussed in Randomised section). In T6, surgeons were able to exclude patients for reasons other than the list of trial protocol inclusion and exclusion criteria, with the reason why recorded as an open response. Two trials (T2, T4) used coded responses to record the reasons for non-trial participation. For example, in T2, the inclusion and exclusion criteria were printed on the back of the screening log, so the research nurse could list multiple reasons why a potential participant was excluded. Two trials (T1, T3) recorded clinical data that could be useful to monitor how key eligibility criteria were applied in different clinical centres.

#### Approached

Two trials did not record any information about approaching patients (T2, T3), which may reflect the assumption that all participants entered into the screening process and log were invited to take part in the trial. Three trials (T1, T5, T6) recorded when written information was provided. Four trials recorded discussions with patients about the trial – either the date of the recruitment appointment (T5, T6) and/or whether the potential participant was approached for consent (Yes/No), (T6, T7, T8). Reasons for not-approaching patients were recorded in five trials, using open responses (T1, T6, T7, T8), and as a subset of codes under ‘reasons for ineligibility’ (T4). Only T6 recorded both written and oral information provision, and reason for not approaching an eligible or potentially eligible participant.

#### Randomised - Final screening outcome and treatment allocation or selection

All screening logs captured information about the final screening outcome and reasons for non-participation. The final outcome of the screening process was most clearly recorded in T1, using four codes - randomised (1), declined (2), ineligible (3), not approached (4). In the next column, an open text box recorded reasons for non-participation, or if enrolled, randomisation ID and treatment allocation. This open text box optimised the use of space to record any reason for non-eligibility (e.g. the clinical result which made the person ineligible); reason for non-approach (e.g. patient not approached in error, patient too unwell to approach); and the reason to decline the trial (e.g. patient did not want treatment X). In this example, it was clear the final column should be filled out for all participants screened.

The quality of data recorded in open text boxes was sometimes limited due to insufficient space, use of open text boxes for multiple answers, and/or it was unclear for which patients the box should be completed. Several trials recorded the reasons why eligible participants declined using three or six coded responses (T2, T5). In addition, T2 recorded the treatment preferences for decliners and final treatment outcome for all screened participants. Five trials (T2, T4, T5, T6, T7) requested information about the final treatment received by non-randomised patients, although this was not always recorded on the screening log.

### The ‘optimised’ SEAR log

**Fig. 1:**
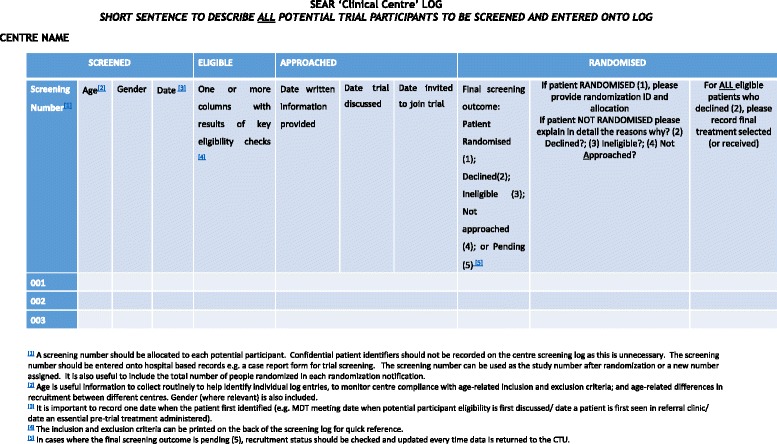
SEAR ‘clinical centre’ log

Results from the content analysis were synthesised to develop an optimised screening and recruitment log to record key aspects of the recruitment pathway. An exemplar ‘clinical centre’ log is shown to demonstrate how data can be captured in a simple format and adapted for use in most trials (Fig. 1), with recommendations for data collection summarised in Table 3. The SEAR framework could also be adapted to collect data using other formats, for example, a CRF-style form or automated database (as in T4, T6, T7).

**Table 3:** Recommended SEAR log checklist

Screened	Eligible
• Develop criteria for entry into the screening process and include instructions on the SEAR log (ideal) • Record basic patient details: age, gender, screening ID, initials (kept locally) • Record basic clinical data for key screening or eligibility criteria as appropriate	• List the inclusion and exclusion criteria on front or back of SEAR log • Note final outcome of eligibility checks against trial inclusion and exclusion criteria • Record reason ineligible using trial inclusion and exclusion codes
Approached	Randomised?
• Date written information provided • Date trial discussed and recruiter initials • Invited to take part in trial (Y/N) • Record reason for non-approach	• Final outcome of screening process (ineligible, not-approached, declined, randomised) • Record reason to decline the trial • Treatment received by eligible patients outside the trial (recorded on screening log or elsewhere as appropriate)

*SEAR* Screened, Eligible, Approached, Randomised

## Discussion

CONSORT guidelines emphasise the importance of recording some basic aspects of screening and eligibility assessment before randomisation, but there has been little consensus over how much data to collect or how best to do this. We compared the content of screening/recruitment logs in eight comparatively small hospital-based RCTs with an integrated QRI which encouraged the keeping of such logs. This content was synthesised to develop a simple framework that could be used or adapted to suit most RCTs, particularly those with recruitment difficulties and multiple clinical centres. The SEAR (Screened, Eligible, Approached, Randomised) framework supports the collection of information that can be used to identify recruitment obstacles which can then be addressed in order to improve the recruitment process.

With so many trials failing to enrol sufficient numbers, recruitment challenges have been well documented – lower than expected numbers of referrals or eligible patients, organisational issues at recruiting centres, problems with the trial design, complex clinical or recruitment pathways, and lack of clinician and patient equipoise [5, 15–18]. Some of these problems could be identified through the collection and analysis of screening data, but few trials collect or publish information about participants before randomisation, beyond the numbers necessary to meet CONSORT guidelines [3, 10]. The SEAR framework augments CONSORT guidelines, and, building on the findings of QRIs, recommends the recording of the number of potential participants excluded because they were not approached, and the reasons why potential participants were excluded from the trial (i.e. reason for ineligibility, non-approach, declined), as well as the final treatment received by eligible patients.

Logging the progress of potential participants through eligibility checks to final decision about trial participation and treatment is relevant to all clinical trials. However methodological interest in screening data has been somewhat limited to emergency medicine trials in relation to concerns about selection bias and the generalisability of trial results [19, 20], in population-based cancer screening studies to increase clinical trial participation [21, 22] and less frequently as a tool to monitor trial eligibility and recruitment rates [23].

The SEAR framework defines four key stages in the recruitment process where participants may be lost, and provides recommendations for data collection. The SEAR framework requires trial-specific adaptations and instructions to harmonise recruitment practices in multicentre trials and optimise the use of resources to identify trial-specific recruitment problems. Unnecessary screening can be minimised through trial-specific criteria for entry into the SEAR process. In practice, the distinction between Screening (initial identification and entry into the SEAR log) and Eligibility assessments (against essential trial inclusion and exclusion criteria), may be blurred in some trials. Time and effort may be wasted screening large numbers of participants who turn out to be ineligible. Other RCTs or clinical centres may take a minimalistic approach to screening, so that only participants deemed highly likely to be eligible are approached to join the trial and entered onto the screening or recruitment log. In trials with small numbers of eligible patients, it may be beneficial to conduct eligibility checks on a larger number of patients to understand key reasons for ineligibility. Although the final outcome of eligibility checks may not be confirmed before patients are approached for trial participation, the SEAR log has been designed to capture information over time (eligibility pending, or awaiting patient decision) and flexibly, according to trial-specific recruitment priorities and concerns.

Least attention has been devoted to define when and how to approach potential participants, although this is where many are often lost [5, 15]. To optimise the number of potential participants approached to join an RCT, we recommend logging the date written information is provided, when the trial is discussed, and when a participant is invited to join the trial. Regarding eligibility, we also recommend data about key eligibility criteria are recorded so that it is possible to identify local decision-making, and final treatment selected or received by non-randomised eligible patients.

Doubts about the scientific value of screening data have led some to argue that the effort to collect high-quality screening data outweighs any benefit [24]. In particular, concerns have been raised about the lack of standardised criteria for *screened* participants and differences in site processes that make the collection of uniform data problematic in multicentre trials. The collection of screening data can be difficult and time-consuming, and may not be needed in detail for all trials, but qualitative research in QRIs has indicated its value. Suboptimal screening can limit the number of potentially eligible patients given the opportunity to enrol in an RCT [25]. Recruiters often struggle to reconcile the desire to gather robust evidence with their clinical instincts and concerns about patient eligibility and safety [5].

Screening data collected by the CTU has been analysed by QRI researchers to help identify these clear obstacles and more subtle hidden challenges so that they can be addressed [15] – for example identifying lower than anticipated numbers of eligible patients and differences between clinical centres in eligibility assessment [10, 26, 27]. Previous research has shown trialists sometimes experience discomfort offering the RCT to all patients, even though the patient technically meets all the trial inclusion and exclusion criteria [5, 15]. Identifying differences in the interpretation of eligibility criteria can be useful to target training and support to improve clinician equipoise [5, 15, 28]. Screening data were also discussed in feedback sessions to address hidden challenges [15, 25], and analysis of final treatment received informed feedback and training sessions to improve equipoise in another trial [26].

There are further examples of the usefulness of log data in the eight RCTs included here. In T1, screening data helped to ensure potential trial participants were identified, logged and provided with information about the trial early in the pathway. Screening practices were particularly important because the condition was rare, and coordination was required across two specialties. Data were also used to identify high volume centres, so that additional training and support could be targeted to improve the recruitment rate in centres screening the most potentially eligible participants. Given greater interest in embedded recruitment studies [29, 30], screening data can provide important information to evaluate the effectiveness of recruitment interventions.

The Screened, Eligible, Approached, Randomised (SEAR) framework supports the collection of data as a tool to map the recruitment process and identify barriers to recruitment. It can be particularly helpful in pilot or feasibility studies, to assess feasibility and inform recruitment strategies for a main trial. Data for the SEAR log can be collected according to the preferred method of the CTU and/ or clinical centres, in the centre log format, using a CRF-style form, or an as app or iPad with supporting database. The SEAR framework and log have been designed so they can be adapted to monitor recruitment in any RCT, but is likely to add most value in trials where recruitment problems are anticipated or evident, and in multicentre trials.

There are some limitations to this study and the framework. The trials included in this study had integrated QRIs, and so there was already a heightened awareness of recruitment issues. The sample size, although small, did include main trials, feasibility, and pilot studies. However, we did not find notable difference between different types of studies. Three out of five pilot/feasibility studies have progressed to a main trial, and are currently using a similar screening log to map the recruitment pathway in the main trial with an integrated QRI. Other trials may find it more difficult to collect such data and it is recommended that the resources needed to support the collection and management of SEAR data are adequately costed for in the grant proposal.

The RCTs sampled here were all National Health Service (NHS) hospital-based studies, evaluating treatments for non-emergency procedures for cancer or chronic conditions. The total number of patients screened for each trial was comparatively small. Much larger trials or trials identifying patients from primary care may need to conduct basic eligibility checks before entering patients into the SEAR process, to streamline the SEAR framework, or may not need it in full. The SEAR process can be time-consuming and requires resources from recruiting centres which may be a problem for some RCTs and CTUs. However, the investment may reveal information about recruitment difficulties that remain such a source of difficulty for many RCTs. The framework could be a useful tool in emergency medicine trials, where it is important to closely monitor the appropriateness and application of eligibility criteria [19, 20].

The SEAR framework and logs included in this study collected information exceeding the CONSORT reporting guidelines, and National Institutes of Health (NIH) (2014) definition of a screening log [11]. However, the CONSORT guidelines conflate screening criteria with eligibility criteria, and eligibility criteria with those approached for study participation. Previous syntheses of qualitative research [4, 5] and findings from the content analysis here show that the recruitment pathway in most trials follows the SEAR stages: Screening, Eligibility, Approached, Randomised. As such, the SEAR framework could be used to update CONSORT reporting guidelines, following further work to test and validate this approach in other types of trials, with different groups of investigators and outside the NHS context. Although CONSORT was extended to improve pilot and feasibility study reporting, the guidelines did not substantially revise data on screening except for reporting numbers approached [31].

## Conclusions

The SEAR framework provides a systematic way to record the flow of potential participants through the recruitment process. It can be adapted to monitor and identify problems in the recruitment pathway of most trials. The reasons and points at which people are ‘lost’ to the trial can be further investigated using qualitative methods or targeted interventions within a QRI to improve recruitment [6]. Data collected using SEAR can also be used to evaluate the QRI or other interventions aimed to improve recruitment. Given that most trials collect some data about potential participants during the recruitment process to meet current CONSORT guidelines, the SEAR framework is a low-intensity intervention to support better recruitment practices in clinical trials, and further work to test it more widely is recommended.

## Abbreviations

CI: Chief investigator
CONSORT: Consolidated Standards of Reporting Trials
CRF: Case report form
CTU: Clinical Trials Unit
GCP: Good Clinical Practice
MDT: Multidisciplinary team
NHS: National Health Service
NIH: National Institutes of Health
NIHR: National Institute of Health Research
QRI: QuinteT Recruitment Intervention
QuinteT: Qualitative research Integrated within Trials
RCT: Randomised controlled trial
SEAR: Screened, Eligible, Approached, Randomised
TM: Trial manager
